# Mapping Postpartum Depression in Latvia: Prevalence and Associated Factors Among Women Receiving Outpatient Care

**DOI:** 10.3390/jcm15030946

**Published:** 2026-01-24

**Authors:** Marija Lazareva, Lubova Renemane, Silvija Cipare, Linda Rubene-Kesele, Vineta Viktorija Vinogradova, Liva Kise, Nancy Byatt, Elmars Rancans

**Affiliations:** 1 Department of Psychiatry and Narcology, Riga Stradins University, LV-1007 Riga, Latvia; 2 Department of Obstetrics and Gynaecology, Riga Stradins University, LV-1007 Riga, Latvia; 3 Departments of Psychiatry and Behavioral Sciences, Obstetrics and Gynaecology, and Population and Quantitative Health Sciences, UMass Chan Medical School, Worcester, MA 01655, USA

**Keywords:** perinatal mental health, postpartum depression, associated factors

## Abstract

**Objectives**: Postpartum depression is a major global mental health concern, yet epidemiological evidence from the Baltic region remains limited. This study aimed to estimate the prevalence of depressive symptoms among postpartum women attending postpartum outpatient care in Latvia and identify associated sociodemographic and clinical factors. **Methods**: A cross-sectional study was conducted at the outpatient department of the largest maternity hospital in Latvia from May 2024 to June 2025. All women aged 18 years or older, who attended a routine postpartum gynaecological visit 4 to 6 weeks after delivery and screened positive on the Patient Health Questionnaire-9 (PHQ-9) (≥5 points), completed a sociodemographic and clinical questionnaire and the Edinburgh Postnatal Depression Scale (EPDS). Descriptive statistics were used in the study, and logistic regression was used to examine factors associated with postpartum depressive symptoms. **Results**: A total of 272 women aged 18 to 49 years (mean age 30.66 ± 5.59) participated. PHQ-9 results indicated that 43.02% of respondents met the threshold for a positive screen (≥5 points) and were included in the further analysis. Using a cut-off EPDS ≥11, the point prevalence of clinically significant depressive symptoms among women who screened positive on the PHQ-9 was 11.4%. In univariate analyses, postpartum depressive symptoms were most strongly associated with comorbid mental disorders (OR = 4.55; 95% CI 1.85–11.18; *p* = 0.001), caesarean section (OR = 3.05; 95% CI 1.18–7.92; *p* = 0.022), stress (OR = 2.49; 95% CI 1.04–5.94; *p* = 0.04) and obstetric complications (OR = 2.78; 95% CI 1.01–7.64; *p* = 0.048) during pregnancy. In the multivariate model, only three independent predictors remained: comorbid mental disorder (aOR = 9.54; 95% CI 2.72–33.49; *p* < 0.001) and caesarean section (aOR = 5.80; 95% CI 1.66–20.21; *p* = 0.006) were associated with higher odds of postpartum depression, while first-time motherhood was associated with a substantially lower likelihood of depressive symptoms (aOR = 0.14; 95% CI 0.04–0.49; *p* = 0.002). Sociodemographic characteristics, including age, education, employment, and income, were not significant predictors. **Conclusions**: The point prevalence of clinically significant depressive symptoms among Latvian postpartum women screening positive for depression appears similar to other European settings. Comorbid mental disorders and caesarean section were the strongest predictors of depressive symptoms, while primiparity showed a protective effect. Sociodemographic factors did not independently contribute to risk. As the first study of its kind in Latvia and conducted within a clinical setting that captures a large and diverse proportion of postpartum women, these findings highlight the context-specific nature of postpartum depression and underscore the need for further longitudinal research to inform effective screening and intervention strategies.

## 1. Introduction

Postpartum depression (PPD), a serious yet often underrecognized condition, is defined as a depressive episode that typically emerges within 4 to 6 weeks after childbirth [1]. Women experiencing PPD may face significant emotional and physical challenges, including overwhelming exhaustion, feelings of worthlessness, and a deep sense of despair [2,3]. Difficulties such as reduced enjoyment of previously meaningful activities, heightened anxiety, and mood fluctuations can substantially diminish a woman’s overall well-being, affecting her sense of self, daily functioning, and quality of life, also make infant care and family interactions more demanding [4].

The prevalence of PPD varies across regions and socioeconomic backgrounds. As of 2021, the prevalence of PPD worldwide, ranged from approximately 15.5% in high-income countries to 19.9% in developing regions, with an overall prevalence rate of 17.22%. Additionally, the study reported regional differences within Europe, estimating the prevalence at 12.91% in Western Europe and 16.62% in Eastern Europe [5]. A recent review found that the pooled prevalence of PPD during the COVID-19 pandemic rose to 34%, nearly twice as high as in the pre-pandemic period [6]. These figures highlight the relevance of PPD as a global mental health concern that transcends cultural and socioeconomic boundaries.

Beyond its negative effects on maternal mental health and physical well-being, postpartum mood disorders can also significantly impact a child’s health. Research suggests that PPD can disrupt mother-infant bonding, which, in turn, may contribute to emotional, social, and cognitive developmental challenges in the child [4,7,8]. Additionally, children of mothers who have experienced PPD tend to exhibit a higher overall morbidity rate [9]. These early developmental struggles can have long-term implications, increasing the risk of behavioural issues, difficulties in school, and emotional instability later in life [4].

Furthermore, when left untreated, maternal depressive disorders are also associated with serious risks, including an increased likelihood of suicidality, as well as higher rates of alcohol and substance use [10,11]. In some cases, PPD has been linked to infanticide, highlighting the urgent need for early detection and intervention [4]. The psychological distress caused by PPD can lead to feelings of hopelessness, causing some women to believe they are unfit to be mothers or that their child would be better off without them [4,12]. This dangerous mindset underscores the critical importance of proper mental health care and strong social support networks for new mothers. Given these far-reaching consequences, it is crucial to identify, understand, and address PPD effectively.

In addition to its clinical implications, PPD presents a significant economic burden for health systems across the globe. Most recently, a large observational study from Germany reported that mothers facing a noticeable mental health or psychosocial burden incurred adjusted total postnatal costs of €10,849, compared to €9136 among mothers without such burden—a difference of €1713 per case [13]. In the UK, lifetime costs of perinatal depression were estimated at £75,728 per affected woman, contributing to a national aggregate cost of £6.6 billion [14]. Other studies say that for a single birth cohort in the United States, total costs reach billions of dollars, including losses in productivity and expenses related to the worsened health of both mother and child [15,16]. These findings reinforce the need for early identification and treatment of PPD—not only to reduce suffering but also to alleviate avoidable economic costs to public healthcare systems.

In recent years, implementing universal screening for depression during the postpartum period has emerged as one of the key approaches to identifying PPD when it occurs [17,18]. When paired with appropriate referral for evaluation and treatment, this strategy can effectively lessen the impact of PPD and help overcome barriers faced by patients, healthcare providers, and the broader health system [19,20]. Various screening instruments are used to detect depression in perinatal populations. Among them, the Edinburgh Postnatal Depression Scale (EPDS) [21] remains the most widely applied tool, and the Patient Health Questionnaire-9 (PHQ-9) [22] is gaining popularity for broader use in large-scale depression screening efforts [18,23].

A wide range of factors that may increase the risk of developing PPD have been identified in studies worldwide. These include socio-demographic factors (e.g., age, education, ethnicity, employment and income level, marital status) [24,25], health-related behaviours such as smoking and alcohol use [26,27], factors related to pregnancy and childbirth [25,28,29], and psychosocial factors (e.g., support from family and friends, exposure to violence, maternal self-esteem, etc.) [25,28,29,30,31].

Despite the global recognition of PPD as an important mental health issue, there is still a limited number of population-based or large-scale studies examining its prevalence and determinants in the Baltic region and Eastern Europe. Currently, Latvia lacks representative and internationally comparable data on the prevalence of PPD and its associated factors. This indicates a significant unmet need and represents a major public health concern. Local studies like ours are essential to improve national health strategies and can be the first step toward addressing PPD. Given the significant impact of PPD on maternal health, child development, and the economic burden it poses, this study aimed to (1) determine the prevalence of clinically significant depressive symptoms among women receiving outpatient postpartum care, and (2) identify the associated sociodemographic and clinical factors. Such understanding is crucial for optimising screening strategies and improving interventions for PPD. It is critical to ensure that all women—regardless of their economic or social circumstances—receive the necessary support to recover and thrive. Additionally, our study carries the potential to lay the groundwork for cross-national analyses, contribute to global mental health databases, and inform the development of culturally responsive screening and diagnostic guidelines.

## 2. Materials and Methods

### 2.1. Study Design and Participants

This cross-sectional quantitative study was conducted in Latvia from May 2024 to June 2025. The study included all postpartum women aged ≥18 years who attended a routine postnatal visit 4–6 weeks after delivery at the outpatient department of Riga Maternity Hospital—the largest maternity outpatient facility in the country, providing care for up to 400 women annually. Women were eligible to participate if they had a PHQ-9 score of ≥5 at their first postnatal gynaecological visit—accordingly, the study was designed to examine depressive symptoms and associated factors among postpartum women at elevated risk.

### 2.2. Sample Size

Assuming a target population size of 400 women at the Riga Maternity Hospital outpatient department, with an expected prevalence of the dependent variable at 16.62% [5] and a traditional Alpha error margin of 5%, the minimum required sample size for this cross-sectional study was 150 women. For an Alpha error margin of 3%, the required sample size increased to 172 women, and for 1%, it was 213 women. The sample size calculation was conducted using the OpenEpi 3.01 calculator [32].

### 2.3. Data Collection

All participants provided written informed consent prior to participation; verbal-only consent was not used. Data were obtained in several ways. The baseline PHQ-9 data was collected during a gynaecological visit at 4–6 weeks postpartum, during which the questionnaire was administered by the consulting gynaecologist. Participants then completed the clinical and sociodemographic questionnaire electronically or responding during a remote interview conducted by the research team. Participants also completed the EPDS electronically. These assessments were completed within two weeks after the initial screening with PHQ-9. Remote data collection was chosen to accommodate the busy schedules of new mothers and to respect their time and primary caregiving responsibilities. General information about the participants’ health status, pregnancy, childbirth, and postpartum medical history was collected from Riga Maternity Hospital medical records.

The study used Latvian and Russian versions of the questionnaires and scales, allowing participants to select their preferred language. To ensure confidentiality, all collected data were anonymized by assigning unique identification numbers to participants, which were subsequently used during data processing, and identifying information was stored separately from research data on secure institutional servers in accordance with data protection regulations. Data storage was securely managed using REDCap, a reliable online platform for survey administration and database management [33]. Licencing and long-term data storage were provided by Riga Stradins University. Access to the database was restricted to authorised study personnel through role-based permissions and password-protected accounts.

### 2.4. Measurements

The medical record data included number of deliveries, history of abortions, pregnancy complications, course and outcome of pregnancy and delivery, results of prenatal diagnostics and genetic screening, complications in previous pregnancies, and history of somatic diseases.

The sociodemographic and clinical questionnaire, developed by the research team, included questions regarding participants’ general demographic data, such as age, place of residence, education, family status, employment, and income level. Additionally, it asked about harmful health-related habits, personal and family history of mental disorders, and experienced stress. The questionnaire was designed based on existing literature on established associated factors for PPD.

In this study, the PHQ-9 served as the threshold tool for participant selection—they completed validated for Latvia Latvian and Russian versions of the questionnaire [34]. It is a self-assessment tool designed to evaluate symptoms of depressive disorders and their severity. This 9-item questionnaire is based on the Diagnostic and Statistical Manual of Mental Disorders, Fifth Edition (DSM-5) criteria for major depressive episode [35]. It is widely used in clinical practice and research to assess depression severity by asking respondents to indicate how often they have experienced symptoms over the past two weeks, thereby evaluating the current depressive episode or point prevalence. In the context of PPD, the PHQ-9 is particularly useful for identifying new mothers experiencing depressive symptoms, allowing for timely intervention and support. The questionnaire assesses core depressive symptoms, including low mood, anhedonia, sleep disturbance, fatigue, concentration difficulties, and appetite, which are commonly affected by PPD. Although it was not specifically designed for PPD, its simplicity and effectiveness make it a valuable screening tool during the postpartum period, demonstrating good reliability and validity in perinatal populations [23,36].

EPDS is a specialised assessment tool designed to identify symptoms of PPD and has demonstrated strong psychometric properties across diverse settings [37,38]. It consists of 10 questions that focuses on emotional and psychological well-being during the postpartum period, addressing aspects such as low mood, anxiety, feelings of guilt or hopelessness, and reduced enjoyment, while minimising confounding somatic symptoms common in the postpartum period [21]. The EPDS is considered to provide greater specificity for PPD compared to other screening tools, such as the PHQ-9 [39,40]. The adaptation of the EPDS into the Latvian language was conducted in a previous Latvian study [37], while the double translation into Russian was carried out by the research team involved in the current study. To evaluate the clarity and comprehensibility of the questionnaire, a pilot study was conducted with 20 women receiving postpartum outpatient care at the outpatient department of Riga Maternity Hospital, integrated as an initial step within the broader framework of this study. Within the main study, the EPDS was used to identify participants with clinically relevant depressive symptoms. The optimal EPDS cut-off for this sample was determined based on the remote diagnostic interview with The Mini International Neuropsychiatric Interview (MINI) 7.0.2 performed by trained psychiatrists within two weeks of screening in a subsample of participants, solely for cut-off calibration and validation purposes. The MINIs were not used for diagnostic confirmation in the full cohort. Based on this calibration procedure, the optimal EPDS threshold was estimated to be ≥11 points, corresponding to a sensitivity of 0.74, a specificity of 0.79, and a Youden index of 0.53. This threshold well aligns with the recent meta-analysis [38], and it was used to classify participants with probable depressive symptoms in subsequent analyses.

### 2.5. Data Analysis

To determine the optimal cut-off point for the EPDS, sensitivity, specificity, positive predictive value (PPV), negative predictive value (NPV), and the Youden index were calculated for a range of possible thresholds. The Youden index (sensitivity + specificity − 1) was used to identify the value that provided the best balance between sensitivity and specificity.

Statistical data analyses were performed using Statistics International Business Machines Corporation’s Statistical Package for the Social Sciences version 29.0 (IBM SPSS 29.0). Descriptive statistics were used in the study, and to examine factors associated with PPD, a hierarchical block-wise regression modelling was performed. Binary logistic regression was applied to estimate odds ratios (OR) in both univariate and multivariate analyses. All results are reported as OR with 95% confidence intervals (CI). A *p*-value of less than 0.05 was considered statistically significant.

Multivariable logistic regression was conducted using a hierarchical block-wise modelling approach with predefined variable groups based on conceptual relevance. The grouping of variables into predefined blocks was determined through interdisciplinary expert discussion involving psychiatrists and gynaecologists, based on clinical relevance and prior evidence. The first group included socio-demographic factors. The second group comprised medical, gynaecological, and perinatal factors extracted from medical documentation. The third group included lifestyle factors, social and emotional variables, and self-reported symptoms reported by participants.

In the first step, all available socio-demographic variables were entered simultaneously into the model. As none of these variables showed a statistically significant association with EPDS ≥ 11 (*p* < 0.05), they were not retained in subsequent steps.

In the second step, medical, gynaecological, and perinatal variables were entered as a block. Variables demonstrating a statistically significant association (*p* < 0.05), as well as one variable showing a near-significant association (*p* ≈ 0.06), were retained for further modelling.

In the third and final step, lifestyle-related, socio-emotional, and self-reported variables were entered together with the retained variables from the second step. Variables not meeting the predefined significance criteria were excluded to reduce model complexity and minimise the risk of overfitting given the limited number of outcome events. This hierarchical approach was applied as an exploratory strategy to assess the robustness of associations while maintaining clinical interpretability.

## 3. Results

Over a 12-month period, data were collected from 272 women aged 18 to 49 years (mean age 30.66 ± 5.59). Based on the PHQ-9 assessment, 43.02% of respondents screened positive (≥5 points) for depressive symptoms and were subsequently included in the further analysis as this tool used as the threshold measure for participant selection. Among eligible women, a proportion (27.59%) declined participation or did not complete follow-up assessments, primarily due to time constraints or loss to contact. According to the EPDS, which was used to identify clinically relevant depressive symptoms, the point prevalence of depressive symptoms within the screened outpatient sample was 11.4% (EPDS ≥ 11). Most participants were aged 31 years or older (51.5%) and lived in the capital city, Riga (75.7%). Among those who completed the detailed sociodemographic questionnaire (n = 101), the majority had higher or incomplete higher education (81.2%) and were married or cohabiting with a partner (97.0%), with only 3% being unmarried or living separately. Most mothers (81.3%) and their partners (94.6%) were employed, while over half of the respondents reported a household income above €1100 or found it difficult to specify. Table 1 summarises the general sociodemographic characteristics of the study sample.

In the univariate analysis, several factors showed statistically significant associations with PPD (defined as EPDS ≥ 11). Women with a comorbid mental disorder had significantly higher odds of PPD (OR = 4.55; 95% CI 1.85–11.18; *p* = 0.001), as did those who had undergone a caesarean section (OR = 3.05; 95% CI 1.18–7.92; *p* = 0.022). Additional factors approaching significance included low infant birth weight (<2500 g) (*p* = 0.051), assisted reproductive technology (*p* = 0.057), and first-time motherhood, which was inversely associated with PPD (OR = 0.45; 95% CI 0.19–1.07; *p* = 0.072). The presence of stress during pregnancy (OR = 2.49; 95% CI 1.04–5.94; *p* = 0.04) and pregnancy complications (OR = 2.78; 95% CI 1.01–7.64; *p* = 0.048) also showed significant associations.

In the multivariate logistic regression hierarchical block-wise modelling, only the most robust predictors remained statistically significant. Comorbid mental disorder remained a strong independent predictor of PPD (adjusted OR = 9.54; 95% CI 2.72–33.49; *p* < 0.001). Caesarean section was also independently associated with increased odds of PPD (aOR = 5.80; 95% CI 1.66–20.21; *p* = 0.006), while being a first-time mother (primipara) was associated with significantly lower odds of developing PPD (aOR = 0.14; 95% CI 0.04–0.49; *p* = 0.002).

Multicollinearity between variables that were significant in univariable analyses but not retained in the multivariable model, was assessed using variance inflation factors (VIF), tolerance statistics, and condition indices. All VIF values ranged from 1.02 to 1.12, tolerance values from 0.89 to 0.98, and the maximum condition index was 10.86, indicating no relevant multicollinearity.

The point prevalence of depression among different groups and results of univariate and multivariate analysis are represented in Table 2. Other variables included in the model (e.g., stress during pregnancy, alcohol use, sleep quality, mood disorders) did not retain statistical significance after adjustment and were excluded from the final model. The complete hierarchical block-wise regression results, including all variables entered at each modelling step, are provided in the Supplementary Materials Table S1.

## 4. Discussion

PPD remains a major global mental-health concern, yet substantial geographical disparities persist in the availability and quality of epidemiological data. This gap is particularly evident in the Baltic region and parts of Eastern Europe, where large, representative, or nationally comparable studies remain scarce. Despite growing awareness of maternal mental health across Europe, much of the evidence informing clinical guidelines is derived from Western European or North American populations, limiting its applicability to countries with different sociodemographic profiles, healthcare systems, and cultural contexts. Against this backdrop, the present study addresses an important regional evidence gap by providing new data on the prevalence and associated factors of depressive symptoms among postpartum women receiving outpatient care in Latvia.

Research from neighbouring Baltic countries demonstrates both notable progress and important ongoing gaps. In Estonia, the most substantial contribution comes from the 2021 study validating the Estonian version of the EPDS, which provided rigorous psychometric evaluation but did not generate population-level prevalence estimates, leaving the broader epidemiological picture unclear [41]. In Lithuania, evidence remains similarly limited: the recently published abstract by Norvaišaitė et al. [42] offers only preliminary cross-sectional results, without detailed methodology, sample characteristics, or validated threshold information, restricting its utility for regional comparison. Beyond the Baltics, studies from Eastern and Central Europe remain limited and heterogeneous. For instance, Finnish data, although population-based in scope, primarily focus on depression during pregnancy rather than the postpartum period—a large national analysis from 2002 to 2010 examined risk factors and perinatal outcomes associated with major antenatal depression, but did not extend to postpartum symptomatology [43]. As a result, the region still lacks robust, nationally representative data that would allow meaningful cross-country comparisons or the development of culturally sensitive screening recommendations.

Taken together, this landscape underscores the importance of generating reliable epidemiological evidence from diverse European contexts. By providing systematically collected, outpatient-based data on PPD prevalence and associated sociodemographic and clinical factors, our study contributes essential knowledge to an under-researched region. These findings provide novel insight into the mental health challenges faced by postpartum women in Latvia and offer valuable data for regional comparison and future meta-analyses, thereby strengthening the broader European evidence base on PPD associated factors [13,44].

Considering these observations, results of our study show that among the sociodemographic characteristics, the majority of participants were over the age of 30, resided in Riga, were of Latvian ethnicity, had higher education, were married and employed or self-employed. While these factors did not independently predict PPD in the regression analysis, they provide important context for interpreting our findings and highlight the relatively advantaged profile of the study population.

In the univariate logistic regression analysis, several variables showed potential associations with PPD. These included stress and complications during pregnancy, caesarean section, and comorbid mental disorders. However, in the final multivariate logistic regression hierarchical block-wise model, only three factors remained statistically significant: presence of comorbid mental disorder, caesarean section, and first-time motherhood (primiparity), which was found to be a protective factor.

The strong association between comorbid mental disorders and PPD aligns with previous research identifying pre-existing or concurrent psychiatric conditions as key predictors of postpartum mental health issues [31,45]. These findings reinforce the importance of comprehensive mental health screening during the perinatal period. Women with a history of depression, anxiety, or other psychiatric conditions may require closer monitoring and tailored support to mitigate the risk of PPD symptoms [46,47,48].

Caesarean section was also independently associated with a significantly increased likelihood of PPD, which is consistent with prior meta-analyses and systematic reviews [47,49,50]. It is plausible that surgical birth, especially when unplanned or emergent, may be experienced as traumatic and contribute to psychological distress, feelings of guilt, or reduced maternal self-efficacy, thereby increasing risk to depressive symptoms. While we did not differentiate between elective and emergency caesarean deliveries, the observed association should be viewed considering this limitation.

Interestingly, being a first-time mother was associated with lower odds of PPD, contrary to some previous studies that have linked primiparity with heightened vulnerability to emotional distress due to lack of experience [51,52]. This discrepancy may reflect context-specific characteristics of the study sample or features of the Latvian postpartum care environment. In Latvia, first-time mothers may receive closer clinical monitoring, more structured postpartum follow-up, and greater informal support from family members, which could buffer psychological distress during the early postpartum period. By contrast, multiparous women may have faced increased stress due to cumulative caregiving responsibilities, increased time demands, and potentially reduced external support, particularly when caring for multiple children. It is also possible that this finding reflects sample-specific factors, including the relatively socioeconomically stable outpatient population studied. These observations suggest that the relationship between parity and PPD is highly context dependent and may be shaped by healthcare structures and social support systems, underscoring the need for further research in diverse settings.

While our study confirmed several well-established associations for PPD, a number of variables frequently reported in the literature did not demonstrate statistically significant associations in our sample. For example, maternal age, which has been identified as a predisposing factor in several studies—especially younger age groups (e.g., <25 years)—did not emerge as significant in either univariate or multivariate analyses. Studies such as Silverman et al. [47] and Bradshaw et al. [44] reported a higher likelihood of depressive symptoms among younger mothers, a pattern they attributed to factors such as limited stability in their careers, unstable relationships, or socioeconomic vulnerabilities. The lack of association in our study could be partially explained by the age distribution of our sample, where the majority were aged over 30 and relatively socioeconomically stable.

Similarly, income level, educational attainment, and employment status have been previously shown to correlate with PPD risk in large population-based studies [24,53], reflecting broader links between socioeconomic disadvantage and maternal mental health. In our analysis, these sociodemographic indicators did not reach statistical significance. This may be due to the relatively homogeneous profile of our participants, many of whom reported higher education, stable employment and residing in the capital city. Recruitment from a single outpatient clinic in an urban setting may have further contributed to limited variability and potential selection bias, thereby reducing the statistical power to detect sociodemographic gradients. Recent empirical evidence suggests that associations between sociodemographic characteristics and PPD are highly context dependent and may vary substantially depending on healthcare access, social support structures, and sample composition, particularly in urban and socioeconomically advantaged populations [54]. These considerations underscore the importance of cautious interpretation of null findings and highlight the need for population-based studies with greater sociodemographic diversity.

Another notable discrepancy is the lack of association between stress during pregnancy and PPD in the final model. While this variable was significant in the univariate analysis, it did not retain significance in the adjusted model. This is somewhat surprising, given the extensive literature linking antenatal stress to postpartum psychological distress [55,56]. It is possible that the effect of stress was mediated or confounded by other factors, such as comorbid mental health conditions, which were retained in the final model.

Furthermore, infant-related factors, including low birth weight, infant health problems, or unplanned pregnancy, have been associated with increased maternal psychological distress in previous studies [57,58,59], but did not reach significance in our analysis. Rather than indicating a lack of relevance, these null findings may reflect the timing and context in which symptoms and associated stressors were assessed. At 4–6 weeks postpartum, transient sleep disruption, adaptive coping processes, and increased external support are common and may attenuate detectable associations at a single assessment point [55]. In addition, maternal psychological distress may be more strongly influenced by subjective perceptions of infant well-being and caregiving demands than by objective infant characteristics alone—dimensions that were not directly assessed in the present study [4]. Notably, both assisted reproductive technology and low birth weight approached significance, suggesting that these associations may become more evident in larger or more heterogeneous samples or later in the postpartum period. Overall, these findings highlight the dynamic and time-sensitive nature of postpartum depressive symptoms and underscore the importance of longitudinal approaches to fully capture the evolving impact of infant-related and behavioural factors.

Beyond regional research gaps, the interpretation of our findings should also consider contextual factors related to the cultural, healthcare, and gender norms specific to Latvia. The Latvian healthcare system provides universal access to maternity and postpartum care, including routine postpartum visits and relatively low financial barriers; however, despite this potential for early identification, no standardised screening for PPD is currently integrated into routine outpatient postpartum care. At the same time, cultural expectations surrounding motherhood—often emphasising maternal resilience, self-reliance, and prioritisation of the child’s needs—may influence both symptom expression and help-seeking behaviours. Informal family-based support, particularly from partners and extended family members, plays an important role in postpartum care and may buffer psychological distress for some women, while potentially masking symptoms in others [4,30]. Gender norms that place primary caregiving responsibility on mothers may further shape stress exposure and reporting patterns. In total, these healthcare and sociocultural factors may partially explain the observed prevalence and associated factors in this study and highlight the importance of interpreting PPD within its broader national and cultural context [44].

Taken together, these differences underscore the importance of considering context-specific variables—including cultural, systemic, and sample-related factors—when interpreting associated factors for PPD. The heterogeneity in findings across studies reflects the multifactorial and complex nature of PPD and calls for tailored screening approaches that go beyond universally applied risk models.

## 5. Limitations

Several limitations should be acknowledged. First, the cross-sectional design of the study precludes causal inferences. Second, the use of self-reported data, including EPDS scores and health history, may have introduced response bias. Third, the analytical sample was restricted to women who screened positive on the PHQ-9 at the routine postpartum visit. While this two-step screening approach reflects clinical practice and facilitated identification of women at higher risk, it may have led to an underestimation of the true prevalence of postpartum depressive symptoms, as women with milder or atypical symptom profiles—potentially better captured by the EPDS—may not have been included. Consequently, the reported prevalence should be interpreted as conservative, and the generalizability of findings to the broader postpartum population in Latvia may be limited. In addition, women accessing outpatient postpartum care may differ systematically from those who do not. Population-based studies without preselection are needed to provide fully representative prevalence estimates.

Additionally, although the initial cohort included 272 women, only a subset of participants completed both screening instruments and met the EPDS ≥ 11 threshold, resulting in a limited number of outcome events. This constrained the complexity of multivariable modelling and increased the risk of statistical instability, wide confidence intervals, and potential overfitting. To mitigate these risks, a hierarchical block-wise modelling strategy with predefined variable groups was applied. Nevertheless, variable selection was partially guided by statistical significance thresholds, which may introduce instability in small samples. Accordingly, the multivariable findings should be interpreted as exploratory and hypothesis-generating rather than confirmatory. Some estimated odds ratios were accompanied by wide confidence intervals, particularly for variables with sparse data or unbalanced categories, likely reflecting limited statistical power rather than true large effects. Larger prospective studies with sufficient event numbers are needed to validate these findings.

## 6. Conclusions

The point prevalence of clinically significant depressive symptoms among postpartum women who screened positive for depression in Latvia falls within the range reported in European clinical and outpatient settings. Additionally, this study offers valuable insight into the factors associated with PPD among women receiving outpatient care in Latvia. While comorbid mental disorders and caesarean section emerged as significant clinical associated factors—and primiparity as a potential protective factor—commonly reported sociodemographic predictors such as age, education, employment, and income were not independently associated with PPD in this sample. These findings underscore the complex and context-specific nature of PPD and point to the limitations of universal risk models. As the first study of its kind in Latvia, this research serves as an important starting point for addressing maternal mental health in the region and highlights the urgent need for more comprehensive, longitudinal, and culturally informed investigations, including qualitative approaches, to better understand the temporal and contextual factors shaping PPD and to inform more effective, context-sensitive screening and intervention strategies.

## Figures and Tables

**Table 1:** Sociodemographic characteristics of the study population among woman attending outpatient department of Riga Maternity Hospital 4–6 weeks postpartum.

Variables		n	%
Age	18–25	52	19.1%
	26–30	80	29.4%
	31+	140	51.5%
Place of residence	Riga	206	75.7%
	Rural area	32	11.8%
	Another city	34	12.5%
Ethnicity ^1^	Latvian	66	24.3%
	Russian	22	8.1%
	Other	13	4.8%
Mother’s education ^1^	Higher and incomplete higher	80	81.2%
	Secondary/incomplete secondary	10	9.9%
	Professional	11	10.9%
Marital status ^1^	Never been married/lived with partner	2	2.0%
	Married, but living separately	1	1.0%
	Married/living with partner	98	97.0%
Mother’s employment	Employed/Self-employed/Other	221	81.3%
	Unemployed	48	17.6%
	Economically inactive	3	1.1%
Partner’s employment	Employed/Self-employed/Other	227	94.6%
	Unemployed	12	5.0%
	Economically inactive	1	0.4%
Income ^1^	≥1100 and difficult to say	55	54.4%
	500–1100	43	42.6%
	No income and <500	3	3.0%

Results are presented for the full baseline cohort (n = 272; n = 240 for partner’s employment). ^1^ Results are restricted to participants who completed the sociodemographic questionnaire developed by the research team (n = 101).

**Table 2:** Point prevalence of PPD among different groups, results of univariate and multivariate logistic regression analysis of variables affecting PPD.

				Univariate Analysis			Multivariable Logistic Regression (Final Block-Wise Model)		
Factor		Depression (EPDS ≥ 11)		OR	95% CI	*p*	aOR *	95% CI	*p*
		n	%						
Sociodemographic factors									
Age	18–25	6	19.3%	0.97	0.31–3.02	0.961			
	26–30	7	22.6%	0.59	0.21–1.64	0.313			
	31+	18	58.1%	1					
Place of residence	Riga	26	83.0%	1					
	Rural area	2	6.5%	0.53	0.10–2.73	0.445			
	Other city	3	9.7%	0.37	0.10–1.39	0.142			
Ethnicity	Latvian	19	61.3%	1					
	Russian	9	29.0%	1.71	0.63–4.67	0.293			
	Other	3	9.7%	0.74	0.18–2.99	0.675			
Mother’s education	Higher and incomplete higher	25	80.6%	1					
	Secondary/incomplete secondary/professional	6	19.4%	0.88	0.31–2.54	0.813			
Marital status	Living with partner	29	93.5%	1					
	No partner	2	6.5%	4.76	0.42–54.56	0.210			
Mother’s Employment	Employed	27	87.1%	1					
	Unemployed and economically inactive	4	12.9%	0.78	0.23–2.68	0.694			
Partner’s employment	Employed	27	96.4%	1					
	Unemployed and economically inactive	1	3.6%	1.19	0.10–13.63	0.892			
Income	≥1100 and difficult to say	13	46.4%	1					
	500–1100	14	50.0%	1.18	0.50–2.79	0.712			
	No income and <500	1	3.6%	1.22	0.10–14.41	0.875			
Medical and psychiatric history									
Somatic illness	No	15	48.4%	1					
	Yes	16	51.6%	1.13	0.49–2.63	0.778			
Gynaecologic illness	No	16	51.6%	1					
	Yes	15	48.4%	1.33	0.57–3.10	0.516			
Comorbid mental disorder	No	11	35.5%	1			1		
	Yes	20	64.5%	4.55	1.85–11.18	**0.001**	9.54	2.72–33.49	**<0.001**
Comorbid prenatal depression	No	30	96.8%	1					
	Yes	1	3.2%	0.80	0.07–7.45	0.802			
Family history of psychiatric illness	No	21	67.7%	1					
	Yes	10	32.3%	1.49	0.59–3.76	0.405			
PMS	No	5	16.1%	1					
	Yes	26	83.9%	1.67	0.55–5.02	0.363			
Pregnancy- and delivery-related factors									
Caesarean section	No	19	61.3%	1			1		
	Yes	12	38.7%	3.05	1.18–7.92	**0.022**	5.80	1.66–20.21	**0.006**
Induced labour	No	12	38.7%	1			1		
	Yes	19	61.3%	1.41	0.60–3.34	0.432	2.14	0.67–6.80	0.199
Complications during/post labour	No	17	54.8%	1					
	Yes	14	45.2%	0.92	0.40–2.16	0.854			
Complications during pregnancy	No	6	19.4%	1					
	Yes	25	80.6%	2.78	1.01–7.64	**0.048**			
Threatened miscarriage	No	27	87.1%	1					
	Yes	4	12.9%	1.93	0.48–7.73	0.355			
Genetic risks	No	30	96.8%	1					
	Yes	1	3.2%	0.36	0.04–3.09	0.348			
Multiple pregnancy	No	30	96.8%	1					
	Yes	1	3.2%	2.3	0.14–38.00	0.561			
Assisted reproductive technology (IVF/IUI/ICSI)	No	26	83.9%	1					
	Yes	5	16.1%	4.30	0.96–19.27	0.057			
History of abortions	No	28	90.3%	1					
	Yes	3	9.7%	3.64	0.58–22.99	0.169			
First time mother (primipara)	No	18	58.1%	1			1		
	Yes	13	41.9%	0.45	0.19–1.07	0.072	0.14	0.04–0.49	**0.002**
Infant-related factors									
Infant weight < 2500 g	No	25	83.3%	1					
	Yes	5	16.7%	4.47	0.99–20.08	0.051			
Infant sex	Female	15	50.0%	1					
	Male	15	50.0%	0.90	0.40–2.22	0.896			
Infant health problems	No	25	80.6%	1					
	Yes	6	19.4%	1.14	0.38–3.38	0.813			
Child temperament	Yes (calm)	15	48.4%	1					
	No (not calm)	16	51.6%	1.56	0.67–3.66	0.305			
Breastfeeding	Yes	24	77.4%	1					
	No	7	22.6%	1.39	0.49–3.95	0.542			
Psychological and psychosocial factors									
Alcohol use ever	No	1	3.2%	1			1		
	Yes	30	96.8%	1.82	0.20–16.97	0.600	1.98	0.14–28.67	0.616
Smoking ever	No	13	41.9%	1			1		
	Yes	18	58.1%	0.82	0.35–1.94	0.648	0.72	0.23–2.25	0.568
Poor sleep quality now (self-reported)	Yes (good)	14	45.2%	1			1		
	No (poor)	17	54.8%	2.06	0.87–4.84	0.100	1.54	0.39–6.02	0.538
Poor sleep quality during pregnancy (self-reported)	No	5	16.1%	1					
	Yes	26	83.9%	1.8	0.60–5.39	0.294			
Mood disorders during pregnancy	No	16	51.6%	1			1		
	Yes	15	48.4%	1.80	0.76–4.25	0.182	1.56	0.53–4.64	0.424
Stress during pregnancy	No	15	48.4%	1			1		
	Yes	16	51.6%	2.49	1.04–5.94	**0.040**	1.59	0.52–4.85	0.417

Statistically significant associations (*p* < 0.05) are marked in bold. * Adjusted odds ratios are based on the final step of the block-wise hierarchical logistic regression model, after sequential exclusion of non-significant variables from preceding blocks.

## Data Availability

The original contributions presented in this study are included in the article. Further inquiries can be directed to the corresponding author.

## Supplementary Materials

The following supporting information can be downloaded at: https://www.mdpi.com/article/10.3390/jcm15030946/s1, Table S1: Factors associated with PPD in the first, second, and third hierarchical block-wise regression models.

## Abbreviations

The following abbreviations are used in this manuscript:

PPD	Postpartum depression
EPDS	Edinburgh Postnatal Depression Scale
PHQ-9	Patient Health Questionnaire-9
DSM-5	Diagnostic and Statistical Manual of Mental Disorders, Fifth Edition
MINI	Mini International Neuropsychiatric Interview
PPV	Positive predictive value
NPV	Negative predictive value
IBM SPSS	International Business Machines Corporation’s Statistical Package for the Social Sciences
OR	Odds ratios
aOR	Adjusted odds ratios
CI	Confidence intervals
PMS	Premenstrual Syndrome
IVF	In Vitro Fertilisation
IUI	Intrauterine Insemination
ICSI	Intracytoplasmic Sperm Injection
VIF	Variance inflation factors
