# Supplementary material for: Mapping Postpartum Depression in Latvia: Prevalence and Associated Factors Among Women Receiving Outpatient Care
Source: J Clin Med. 2026 Jan 24;15(3):946. doi: 10.3390/jcm15030946 (PMC12898396; doi:10.3390/jcm15030946)
Supplement: Supplementary file 1 [file jcm-15-00946-s001.zip › jcm-4062023-supplementary.pdf]

Table S1. Factors associated with PPD in the first, second, and third hierarchical block-wise regression models.

Factor		aOR1	95% CI	p	aOR2	95% CI	p	aOR3	95% CI	p
Proximal factors										
Sociodemographic factors										
Age	18–25	0.98	0.23–4.07	0.974						
	26–30	0.64	0.21–1.99	0.443						
	31+	1								
Place of residence	Riga	1								
	Rural area	0.78	0.13–4.89	0.794						
	Other city	0.18	0.04–0.97	0.046						
Ethnicity	Latvian	1								
	Russian	2.26	0.68–7.54	0.186						
	Other	0.78	0.17–3.64	0.749						
Mother's education	Higher & incomplete higher	1								
	Secondary / incomplete secondary / professional	0.59	0.15–2.32	0.452						
Marital status	Living with partner	NE								
	No partner									
Mother's Employment	Employed	1								
	Unemployed & economically inactive	1.43	0.34–6.08	0.625						
Partner's employment	Employed	1								
	Unemployed & economically inactive	1.39	0.09–21.52	0.812						
Income	≥1100 & difficult to say	NE								
	500–1100									
	No income & <500									

**Intermediate factors**										
Medical and psychiatric history										
Somatic illness	No				1					
	Yes				1.58	0.40–6.17	0.512			
Gynaecologic illness	No				1					
	Yes				2.21	0.48–10.17	0.308			
Comorbid mental disorder	No				1			1		
	Yes				21.22	3.33–135.34	**0.001**	9.54	2.72–33.49	**<0.001**
Comorbid prenatal depression	No				1					
	Yes				0.03	0.00–1.94	0.100			
Family history of psychiatric illness	No				1					
	Yes				0.87	0.15–4.92	0.872			
PMS	No				1					
	Yes				1.44	0.28–7.31	0.659			
Pregnancy- and delivery-related factors										
Caesarean section	No				1			1		
	Yes				8.83	1.28–60.73	**0.027**	5.80	1.66–20.21	**0.006**
Induced labour	No				1			1		
	Yes				4.30	0.93–19.77	**0.061**	2.14	0.67–6.80	0.199
Complications during/post labour	No				1					
	Yes				0.77	0.16–3.61	0.735			
Complications during pregnancy	No				1					
	Yes				2.79	0.50–15.46	0.241			
Threatened miscarriage	No				1					
	Yes				2.44	0.17–35.41	0.513			
Genetic risks	No				1					
	Yes				0.06	0.00–3.72	0.179			
Multiple pregnancy	No				1					
	Yes				0.68	0.01–80.19	0.872			

Assisted reproductive technology (IVF/IUI/ICSI)	No				1					
	Yes				2.60	0.28–24.68	0.404			
History of abortions	No				1					
	Yes				14.60	0.31–683.14	0.172			
First time mother (primipara)	No				1			1		
	Yes				0.10	0.02–0.57	**0.009**	0.14	0.04–0.49	**0.002**
Infant-related factors										
Infant weight <2500 g	No				1					
	Yes				10.82	0.62–189.08	0.103			
Infant sex	Female				1					
	Male				1.62	0.41–6.35	0.491			
Infant health problems	No				1					
	Yes				3.99	0.39–40.95	0.244			
Child temperament	Yes (calm)				1					
	No (not calm)				2.37	0.45–12.55	0.311			
Breastfeeding	Yes				1					
	No				1.35	0.20–8.98	0.758			
**Distal factors**										
Psychological and psychosocial factors										
Alcohol use ever	No							1		
	Yes							1.98	0.14–28.67	0.616
Smoking ever	No							1		
	Yes							0.72	0.23–2.25	0.568
Poor sleep quality now (self-reported)	Yes (good)							1		
	No (poor)							1.54	0.39–6.02	0.538
Mood disorders during pregnancy	No							1		
	Yes							1.56	0.53–4.64	0.424

Stress during pregnancy	No							1		
	Yes							1.59	0.52–4.85	0.417

Statistically significant associations ($p < 0.05$) and trends ($0.05 \leq p < 0.10$) are shown in bold.

NE – adjusted odds ratio could not be estimated due to small subgroup size.
